# The Bedford Mineral Springs Co.

**Published:** 1894-09

**Authors:** 


### THE BEDFORD MINERAL SPRINGS CO.

Through an arrangement made with Jos. P. Reed, of Philadelphia, we ran an advertisement one year, of the above company.

Having received nothing for the work, and having been unable to secure a settlement, we deem it proper to warn all who may have or expect to have, dealings with them, to demand cash in advance. We do not know whether *Reed* or the *Company* is to blame.

